# A validated HPLC-MS/MS method for simultaneously analyzing curcumin, demethoxycurcumin, bisdemethoxycurcumin, tetra-hydrocurcumin and piperine in human plasma, urine or feces

**DOI:** 10.1016/j.heliyon.2023.e15540

**Published:** 2023-04-20

**Authors:** M.A.G.M. Kroon, H.W.M. van Laarhoven, E.L. Swart, E.M. Kemper, O. van Tellingen

**Affiliations:** a Department of Pharmacy and Pharmacology, Amsterdam UMC Location AMC, the Netherlands; b Department of Medical Oncology, Cancer Center Amsterdam, Amsterdam UMC, University of Amsterdam, the Netherlands; c Department of Pharmacy and Pharmacology, The Netherlands Cancer Institute-Antoni van Leeuwenhoek Hospital, Amsterdam, the Netherlands

**Keywords:** Curcumin, Curcuminoids, Tetrahydrocurcumin, Piperine, HPLC-MS/MS, β-glucuronidase, Human plasma, Human urine, Human feces

## Abstract

**Background:**

The spice curcumin is supposed to have many different beneficial health effects. To understand the complete pharmacokinetics of curcumin we need an analytical method to determine curcumin and its metabolites in human plasma, urine or feces. We have developed an HPLC-MS/MS method for the simultaneous analysis of curcumin, demethoxycurcumin, bisdemethoxycurcumin, tetrahydrocurcumin and piperine in human plasma, urine or feces.

**Methods:**

Sample pretreatment involved a simple liquid-liquid extraction with *tert*-butyl methyl ether. Conjugated curcumin and analogs can be measured after enzymatic hydrolysis. Reversed-phase chromatography with a linear gradient of 50–95% methanol in 0.1% formic acid was used. Total run time is 15 min. The method was validated with regards to stability, specificity, sensitivity, linearity, accuracy, repeatability and reproducibility. The applicability of the method was tested using actual patients samples.

**Results:**

The LLOQ in plasma, urine and feces for curcumin, demethoxycurcumin, bisdemethoxycurcumin, tetrahydrocurcumin and piperine ranged from 1 to 5 nM. Whereas all compounds could be quantified on a linear range between 2 and 400 nM. Plasma and feces recovery of curcumin was 97.1 ± 3.7% and 99.4 ± 16.2%, whereas urine showed a recovery of 57.1 ± 9.3%. All compounds had acceptable in-between day or between day variability in the different matrixes.

**Conclusion:**

A HPLC-MS/MS method was developed and validated for the simultaneous quantification of curcumin, demethoxycurcumin, bisdemethoxycurcumin, tetrahydrocurcumin and piperine in human plasma, urine or feces. This method will aid in critically verifying the pharmacokinetics of curcumin made by supplement manufacturers and help us to provide insight in the claimed bioavailability of curcumin supplements.

## 1 Introduction

**Fig. 1:**
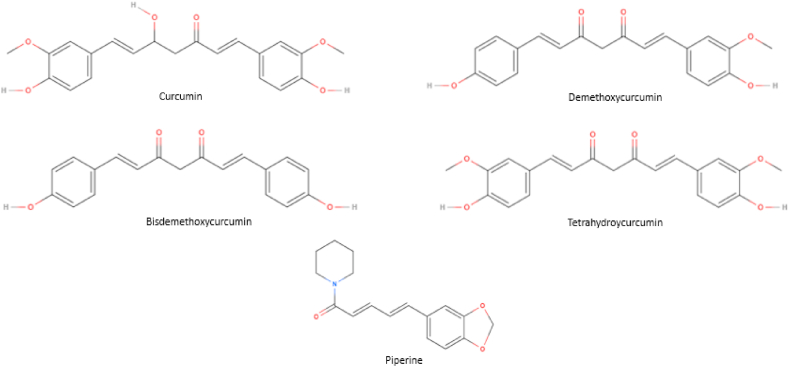
Molecule structures of curcumin, demethoxycurcumin, bisdemethoxycurcumin, tetrahydrocurcumin and piperine.

The spice kurkuma originates from the root of the *Curcuma Longa* and has been used for centuries in Asian cultures as dietary pigment, spice and traditional medicine. Kurkuma consists of curcuminoids, namely curcumin, demethoxycurcumin and bisdemethoxycurcumin with their molecule structures displayed in Fig. 1. Numerous claims regarding the presumed beneficial health effects of curcumin as anti-inflammatory, anti-cancer, anti-Alzheimer agent have been reported [[1], [2], [3]]. Studies conducted in vitro usually examine the effects of curcumin in the micromolar range. For example, curcumin showed to decrease ROS concentration and TNF-α release in retinal pigmented epithelial cells and retinal endothelial cells at a concentration of 10 μM [4]. Furthermore, SW620 cells incubated for 2 days with 10 μM curcumin showed significantly downregulated phosphorylation levels of ERK and STAT1 [5]. However, although many clinical studies have been conducted, none of these have convincingly shown clinical benefit [6]. A Phase II study in patients with pancreatic cancer described some anecdotal beneficial effect in only two out of 21 treated patients [7]. Nevertheless, the storm of reviews have spread to the general public and made curcumin into a popular agent for self-medication.

The lack health effects by curcumin may in part be caused by its poor oral bioavailability. Importantly, curcumin contains an hydroxyl group that is readily conjugated with glucuronide or sulfate, allowing high first-pass metabolism. To improve this low bioavailability of curcumin, supplement manufacturers have developed alternative formulations, like lipid and colloidal formulations [8]. Multiple clinical trials on curcumin nanoformulations are currently registered on clinicaltrials.gov [9]. Next to these new lipid based formulations, it has been suggested that the addition of certain adjuvants could improve the bioavailability of curcumin. One clinical study showed that addition of 20 mg/kg piperine increased bioavailability of curcumin with respectively 154% in rats and 2000% in humans [10]. Piperine is supposed to inhibit the phase I and II metabolism by inhibition of CYP3A4 and iUDP-glucuronosyl transferase (UGT) and thereby increasing the bioavailability of curcumin [[11], [12], [13]].

In order to assess the validity of these claims on improved oral bioavailability and to conduct mass balance studies, a method in which curcumin can be measured in different bodily fluids is needed. As yet, several high performance liquid chromatography – ultra violet (HPLC-UV) or – tandem mass spectrometry (HPLC-MS/MS) methods of curcumin in plasma have been described, however there has not been any HPLC-MS/MS method described that could simultaneously analyze curcumin, curcumin analogs and piperine in human plasma, urine and feces [[14], [15], [16]]. Most of the published articles describe high concentrations of unconjugated form of curcumin [[17], [18], [19]]. Howbeit, this has been achieved after the addition of β-glucuronidase to the sample. This hydrolysis the glucoronidated and sulphated metabolites of curcumin resulting in increased levels of unconjugated curcumin. The samples in this paper were prepared in a parallel procedure where samples were pre-treated with and without hydrolysis of the glucuronidated- and sulphated metabolites.

The objective of this paper is to develop a validated, sensitive method for the quantification of curcumin, demethoxycurcumin, bisdemethoxycurcumin, tetrahydrocurcumin and piperine in human plasma, urine or feces. The compounds of interest were validated on stability, specificity, sensitivity, linearity, accuracy, repeatability and reproducibility. Furthermore, the applicability of the method was tested in samples obtained from participants using a curcumin supplement.

## 2 Materials and methods

### 2.1 Materials and reagents

The reference standards curcumin (≥98% HPLC), demethoxycurcumin (≥98% HPLC), bisdemethoxycurcumin (≥98% HPLC), piperine analytical standard and β-glucuronidase from *Helix pomatia-*type H-1 (partially purified powder) were purchased from Sigma-Aldrich (St. Louis, MOI, USA), The Netherlands). Tetrahydrocurcumin, and the Internal Standards (IS) curcumin-D6, (2E)-demethoxycurcumin-d7, (E,E)Bisdemethoxycurcumin-D8, Tetrahydrocurcumin-D6 and Piperine-D10 were purchased from Toronto Research Chemicals (Toronto, Canada). Dimethylsulfoxide (DMSO) was purchased from Merck (Darmstadt Germany), formic acid was purchased from Sigma Aldrich and, Methanol absolute (MeOH) and *tert*-butylmethyl ether (TBME)) was purchased from Biosolve BV (Valkenswaard, The Netherlands). Milli-Q water was obtained with an Elga Medico Pro watersystem. Blank human plasma (Omniplasma® (Octapharma GmbH, Langenfeld, Germany)) was used to prepare calibration and quality control (QC) samples. Omniplasma is a product produced from pooled Dutch obtained human plasma which underwent a solvent detergent ion and prion reduction step. Single blank human urine and feces samples were obtained from a healthy volunteer that followed a restriction diet for 5 days in which any products containing curcumin and piperine were avoided.

### 2.2 Optimization

The method was optimized on the following parameters: Sample preparation (type of extraction, extraction solution, reconstitution solution composition), column type, mobile phase composition resulting in a gradient formation and injection volume. For the measurement of hydrolysis by glucuronide and sulfate metabolites, the hydrolysis method was optimized based on measurement of curcumin and curcumin-β-glucuronide. This last compound was not included into the validation of the method but was used as an indication for complete hydrolysis of the metabolites. Just as in *in vivo* samples, multiple glucuronide-as well as sulfate metabolites exists. We did not validated this method for a single glucuronide metabolite but used these compounds as indicative measurements for validating the hydrolysis method by β-glucuronidase [20].

### 2.3 Instrumentation

A Thermo Scientific Savant Speedvac high capacity concentrator in combination with a Thermo Scientific Savant Refrigerated Vapor Trap was used for evaporation under vacuum of the samples during sample pretreatment. An ultrasonic cleaner from Branson was used for reconstitution and homogenization of the residue.

The HPLC-MS/MS system consisted of an Ultimate 3000 autosampler and pump with inline degasser, all of Dionex (Sunnyvale, CA, USA), connected to a degasser from LC Packings. The autosampler with a 100 μL sample loop was coupled to an AB Sciex API4000 mass spectrometer (Framingham, MA, USA) via a Shimadzu Prominence Column Oven (type CTO-20A) which was kept at 35 °C. Separation was performed with an Agilent column 2.1 × 100mm packed with material of Zorbax Extend 3.5 μm C-18 and kept at 35 °C using a CTO-20A column oven (Shimadzu, Kyoto, Japan). Instrument control and data acquisition was done using Analyst v1.6.2 and DCMS-link v2.12 software (Dionex).

### 2.4 HPLC-MS/MS conditions

After optimization and stability results (see section 3.8) the chromatographic separation was performed with an injection volume of 50 μL and the autosampler set at 10 °C. The flowrate was 200 μL/min and the dual gradient mobile phase consisted of A: purified H_2_O with 0.1% formic acid and B: MeOH 100%. The applied gradient profile started at 50:50 A: B and increased to 95% B in 3.0 min. During 6 min a 5:95 A:B level was continued, after which it returned in 0.2 min–50:50. Afterwards the system was equilibrated during 6 min at the starting level. Electrospray ionization in positive mode was used as ion source in the MS/MS. The following standard values were used for all runs; collision gas: 4 psig, curtain gas: 30 psig, Ion Source Gas 1: 50 psig, Ion source Gas 2: 50 psig, IonSpray Voltage: 5500 V, Temperature: 400 °C. The mass of the individual fragments in Q1 and Q3 with corresponding Collision Energy (CE), Declustering Potential (DP), Entrance Potential (EP), Collision Cell Potential (CCP), Counts per Second (cps) were set according to Table 1. Multiple reaction monitoring (MRM) mode was used for detection using a dwell time of 100 ms.

**Table 1:** Mass and voltage per compound. CE=Collision Energy, DP = declustering potential, EP = Entrance potential, CXP = collision cell potential, cps = counts per second.

	Q1 Mass (Da)	Q3 Mass (Da)	CE (Volts)	DP (Volts)	EP (Volts)	CxP (Volts)	Intensity (cps)
Curcumin	369.4	177.1	25	55	11	12	2.4E5
Curcumin-D6	375.2	145.2	45	52	9	10	5.5E5
Demethoxycurumin	399.1	119.0	53	60	9	8	1.5E5
Bisdemethoxycurcumin	309.4	199.3	50	55	8	8	3.8E4
(E,E)bisdemethoxycurcumin-D8	317.3	123.1	48	60	10	8	4.5E5
Tetrahydrocurcumin	373.2	137.2	35	70	10	9	3.2E5
Tetrahydrocurcumin-D6	379.4	140.1	37	80	11	10	1.8E5
Piperine	286.2	115.2	60	85	9	7	1.6E6
Piperine-d10	296.4	115.2	65	100	9	8	1.3E6

### 2.5 Analytical procedures

#### 2.5.1 Preparation of stock solutions, calibration- and quality control standards

Stock solutions of each individual compound and each internal standard (IS) were made at concentration of 10 mM in DMSO. For each IS an intermediate stock solution of 1 mM was prepared. A mixed stock solution containing each compounds (compound-mix) and a mixed stock IS solution (IS-mix) were prepared at a concentration of 100 μM in DMSO. Stock solutions of curcumin-β-D-glucuronide 10 mM and 100 μM in DMSO were prepared. All stock solutions were stored at −20 °C and protected from light. A working solution of IS-mix at 100 μM in MeOH: H_2_O 60%:40% was stored at −20 °C until use. Compound-mix calibration standards with concentrations of 0.2 μM, 0.5 μM, 1 μM, 2 μM, 5 μM, 10 μM, 20 μM and 40 μM and compound-mix quality control (QC) standards with concentrations of 2 μM, 5 μM and 10 μM were prepared each day of sample analysis. Calibration and QC samples were prepared by adding 5 μL of calibration or QC standard to 495 μL blank omniplasma. Each sample was processed and measured in duplicate. β-glucuronidase solution was freshly prepared at 20 U/μL by adding sodium-acetate solution (pH 5.0). At the start of every run the system suitability was tested by preparing a mixture of known concentrations of IS and curcumin in 60%:40% (MeOH: H_2_O).

#### 2.5.2 Sample pre-treatment

The optimized method resulted in a sample preparation where 50 μL IS-mix 100uM was added to 100 μL human plasma, urine or diluted feces sample. One mL TBME was added to the mixture. Feces samples were first diluted 10.000x (four steps of 1:10 dilution) with Bovine Serum Albumin (BSA) 2% in Milli-Q water to reduce matrix effects before 50 μL IS-mix 100uM was added to 100 μL feces dilution. A second 100 μL sample with IS solution was hydrolyzed with 50 μL 20 U/μl β-glucuronidase, vortexed and after this addition incubated for 1 h at 37 °C. Following 1 mL TBME was added. After shaking for 15 min at 480 rpm/min all samples were centrifuged at 18213×*g* during 1 min. The lower aqueous layer was frozen on dry-iced ethanol and the upper organic layer of approximately 1 mL was decanted into a fresh tube and dried under vacuum using a Speedvac at room temperature. Next, samples were reconstituted with 100 μL MeOH:H_2_O 60%:40%, vortexed and sonicated for 5 min. After centrifuge at 18213×*g* during 3 min the supernatant was ready for injection into the HPLC-MS/MS.

#### 2.5.3 Quantification

Peak areas were used for quantification of all compounds except tetrahydrocurcumin, which was quantified using peak heights. Internal standard method was used. Each compound had its own deuterated stable isotope labeled analog, except demethoxycurcumin for which (E,E)Bisdemethoxycurcumin-D8 was used. Concentrations were calculated on basis of the peak area or height – for tetrahydrocurcumin – ratio between compound and IS. The compound/IS ratios of unknown samples were back calculated on basis of the calibration curve. The weight distribution (1/x or 1/x^2) was determined for each individual compound calibration curve in each separate matrix.

### 2.6 Method validation

Internal validation protocols based on the FDA Bioanalytical Method Validation guidance for industry, version May 2018, were followed for the following parameters in human plasma: selectivity, specificity, limit of detection (LOD), limit of quantification (LOQ), linearity, recovery and ionization, efficiency, accuracy, within day and between day precision and stability. After completion of the validation in human plasma, the method was validated in urine and feces following a limited validation protocol measuring recovery, ion suppression, selectivity, specificity and accuracy. Statistical analysis was performed using SPSS version 26.

#### 2.6.1 Selectivity, specificity and LOD

Blank urine and feces of three different individuals and three different batches of pooled blank plasma were analyzed for presence of interferences in duplicate in three runs. Quality Control (QC) samples of 2, 5 and 10 nM were measured in duplicate in the same three runs in plasma, urine and feces. Pooled plasma, urine and feces measurements of the calibration curve were used (N = 2) during three runs. The LOD was set at a signal to noise >3, as the ratio between measured peak height of the compound and signal height of the blank.

#### 2.6.2 Lowest limit of quantification and Upper Limit Of Quantification

QC samples of 1, 2, 5, 50, 100, 200 and 400 nM (N = 6 per concentration) were measured in plasma, urine and diluted feces during three runs to determine the Lowest Limit Of Quantification (LLOQ) and Upper Limit Of Quantification (ULOQ). The relative standard deviation within each individual run and the percentage difference between measured concentration and nominal value should be <20%.

#### 2.6.3 Linearity

A new calibration curve was measured each day during following three days – using the concentrations described in section 2.5.1 – and for each calibration curve the power value was established by weight estimated regression analysis in SPSS. This is executed for plasma as well as for pooled omniplasma, urine and feces. The mean power value was used to determine the optimal weighing factor for linear regression. Weighted linear regression with ANOVA was performed to calculate the F Lack-of-Fit (F_LOF_) for each individual line. If the F_LOF_ is below the reference value (F_0,05_) there is no “Lack of Fit” and the calibration curve is linear. As lowest value for the calibration curve the LOQ was used. The highest concentration of linearity was calculated for each individual compound and run. If the line was linear between LOQ and a concentration in two or three out of three runs, this concentration was set as the upper boundary of linearity. For the linear line the systematic and proportional error was established by weighted linear regression analysis with data on the measured concentrations (obtained with Analyst) and the nominal concentration all three days.

#### 2.6.4 Recovery and matrix effect

Plasma, urine and feces were spiked with a compounds-mixture at 8 concentrations *prior* to sample pretreatment and assayed in duplicate. A second set of blank plasma, urine and feces was pretreated and reconstituted using analytical standards of MeOH:water 60%:40% containing the same range of concentrations of compounds-mixture. These calibration curves were made and analyzed 3 times during 3 different runs together with the unprocessed calibration curves. The recovery of the sample pretreatment was calculated from the ratio of the slopes of the *pre*-pretreatment spike over the *post*-pretreatment spiked samples. Any matrix effect by the biological matrix was calculated from the ratio of the slopes of the *post*-pretreatment spike over the unprocessed calibration curve.

#### 2.6.5 Accuracy and precision

Six QC samples per concentration (10, 50, 100 or 200 nM) were prepared in omniplasma, urine and diluted feces and measured together with a calibration curve during three different runs on different days. The average and 95%-confidence interval (95%CI) for each concentration was calculated with SPSS. The accuracy was determined by dividing the mean observed concentration by the nominal concentration and should be within 85% and 115%. Within and between-day precision were calculated using one-way ANOVA analysis with SPSS. The root of mean square within groups was divided by the total mean to obtain the within-day precision. The between-day precision was calculated by dividing the difference between mean square between groups by mean square within groups by six, the root of this number was divided by the total mean. For the lowest concentration the within- and between-day precision should be <20% and for the other concentrations <15%.

#### 2.6.6 Stability

Freeze-thaw stability of QC stock solution 10 μM at −20 °C was performed with stock solution that had underwent 0, 1 and 10 freeze-thaw cycles. Three samples per freeze-thaw cycle in MeOH:H_2_O 60%:40% were prepared at 100 nM and measured in duplicate. Four QC samples per concentration were prepared in plasma. Four replicates per sample were frozen for 60 min at −20 °C. All samples were thawed at room temperature and frozen ones (cycle 1). Per four replicates and concentrations the samples were thawed and frozen further a second, third or fourth time. All samples were thawed at the same time for simultaneous assessment with a calibration curve.

### 2.7 Applicability of the method

Data from three healthy male participants included from a study researching the bioavailability of curcumin formulations is presented to show the applicability of the method in real-life settings. The participants signed informed consent and followed diet restrictions to avoid any curcumin or piperine intake five days before the visit day to ensure that only curcumin and/or piperine from the curcumin capsules were observed. On the visit day a plasma concentration curve at t = 0, 0.25, 0.50, 0.75, 1, 1.5, 2, 4, 8 and 24 h, collection of 24-h urine and 48-h feces were taken after single oral intake of 4 commercially available soft capsules containing 600 mg curcuminoids (curcumin, demethoxycurcumin and bisdemethoxycurcumin with 95% purity). The trial has been approved by the local institutional ethics committee and has been registered in the Dutch Clinical Trial Register under registration number NL6552. Each individual sample was analyzed according to the method described above.

## 3 Results

### 3.1 Tuning of the mass spectrometer

Each individual compound and corresponding internal standard was tuned and the optimized results are presented in Table 1.

### 3.2 Chromatography

**Fig. 2:**
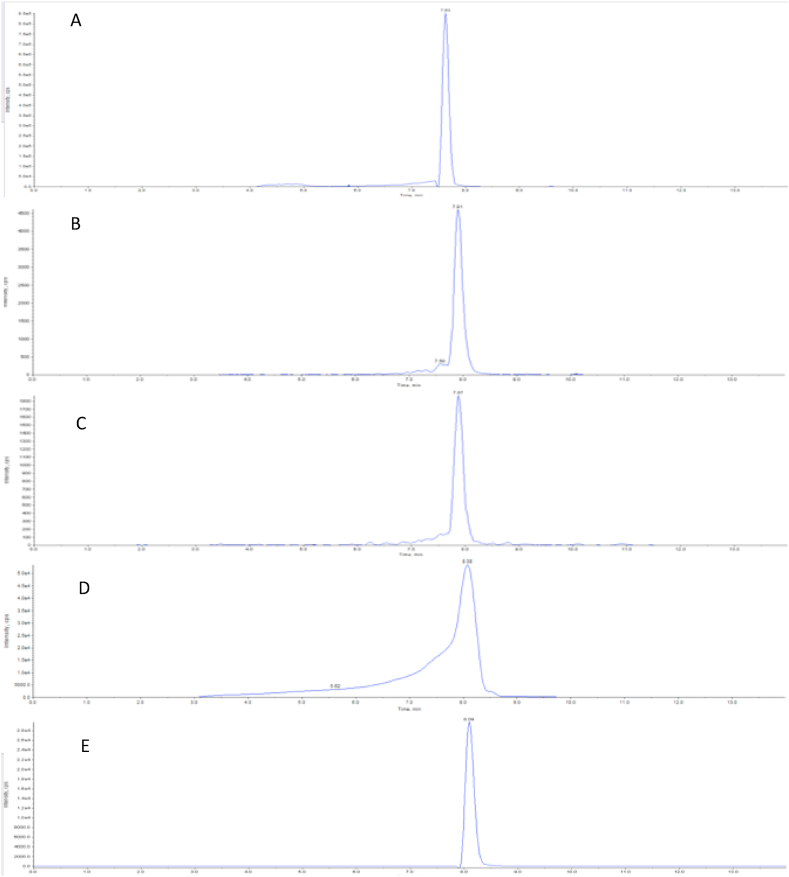
Chromatograms of curcumin (A), demethoxycurcumin (B), bisdemethoxycurcumin (C), Tetrahydrocurcumin (D) and piperine-135 (E). Curcumin elutes at 7.6 min whereas demethoxycurcumin elutes at 7.9 min, bisdemethoxycurcumin at 7.9 min, tetrahydrocurcumin at 8.1 min and piperin-135 elutes at 8.1 min. Tetrahydrocurcumin shows significant front tailing due to the acidic mobile phase.

**Fig. 3:**
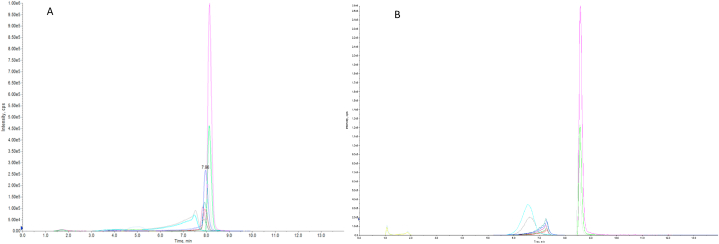
Overview of curcumin, curcumin-d6, demethoxycurcumin, bisdemethoxycurcumin, (E,E)bisdemethoxycurcumin-D8, tetrahydrocurcumin, tetrahydrocurcumin-D6, piperine and piperine-D10 at 100 nM in omniplasma with (A) 0.1% Acetronil as mobile phase and (B) 0.02% ammoniumhydroxide as mobile phase. All compounds and internal standards elutes between 7 and 8 min. (A) whereas this was around 6–7 min in the basic mobile phase conditions (B).

The chromatographic results after injection of 100 nM curcumin, demethoxycurcumin, bisdemethoxycurcumin, tetrahydrocurcumin and piperine are depicted in Fig. 2, Fig. 3. Fig. 2 shows all individual compounds. Curcumin (Fig. 2A) eluted of the column at approximately 7.6 min, demethoxycurcumin (Fig. 2B) at approximately 7.9 min, bisdemethoxycurcumin (Fig. 2C) at approximately 7.9 min, tetrahydrocurcumin (Fig. 2D) at 8.1 min and piperine (Fig. 2E) eluted at approximately 8.1 min. Tetrahydrocurcumin exerts significant front tailing, which appears to be due to the acidic mobile phase. Fig. 3A shows the chromatograms of all compounds including their deuterated internal standards. Fig. 3B shows the chromatograms of all compounds including their deuterated internal standards with 0,02% ammonium hydroxide as mobile phase.

### 3.3 Selectivity, specificity and LOD

The same samples used in determining the selectivity and specificity were used as in calculation LOD (Table 2). No carry over was found in any of the matrixes since there was no curcumin (analog) peaks in blank samples. All compounds had a signal-to-noise ratio greater than 3. This means that all compounds can be quantified at a minimum concentration of 2 nM, except for bisdemethoxycurcumin which had a signal-to-noise ratio at 2 nM lower than 3. Therefore, the LOD of bisdemethoxycurcumin was 5 nM.

**Table 2:** Selectivity and specificity of curcumin, demethoxycurcumin, bisdemethoxycurcumin, tetrahydrocurcumin and piperine-135 in human plasma. The LOD is set at a signal to noise >3, as the ratio between measured peak height of the compound and signal height of the blank.

Compound	Plasma			Urine			Feces		
	Average peak height blank (cps)	Average peak height (cps)	Signal/Noise	Average peak height blanc (cps)	Average peak height (cps)	Signal/Noise	Average peak height blanc (cps)	Average peak height (cps)	Signal/Noise
Curcumin (2 nM)	26.3	247.5	9.4	85.5	5250	61.4	33.7	3675	109
Demethoxy-curcumin (2 nM)	9.8	36.2	3.7	64.4	409.0	6.35	21.3	1152	54.1
Bisdemethoxy-curcumin (5 nM)	6.4	50.1	7.9	44.0	307.6	6.98	21.1	1388	65.9
Tetrahydro-curcumin (2 nM)	2.7	31.9	11.7	37.4	652.7	17.5	72.6	743	10.2
Piperine-135 (2 nM)	31	365.2	11.8	3218	5840	1.81	266	5894	22.1

### 3.4 LLOQ and ULOQ

To determine the LLOQ, three quality control samples (1 nM, 2 nM and 5 nM) were analyzed and to determine the ULOQ three quality control samples (50 nM, 100 nM and 200 nM for urine samples and 100 nM, 200 nM and 400 nM for plasma and feces samples) were analyzed in six-fold (Table 3). A different quality control concentration for urine was chosen due to curcumin metabolism resulting in lower expected concentrations of curcumin, demethoxycurcumin and bisdemethoxycurcumin in urine. The same reasoning applies to quality concentrations in plasma and feces of biological samples. Following these results the LLOQ in plasma for curcumin, demethoxycurcumin, bisdemethoxycurcumin, tetrahydrocurcumin and piperine was 2 nM, 2 nM, 5 nM, 1 nM and 2 nM, respectively. The LLOQ in urine was 2 nM, 2 nM, 5 nM, 2 nM and 5 nM, respectively. The LLOQ in feces was validated on 2 nM, 1 nM, 5 nM, 1 nM and 2 nM, respectively.

**Table 3:** Quantification results of determining the LLOQ and ULOQ of curcumin, demethoxycurcumin, bisdemethoxycurcumin, tetrahydrocurcumin. RSD = relative standard deviation, DEV = standard devation, n.d. = not determined. Three QC monsters (1 nM, 2 nM and 5 nM) and three QC monsters for urine samples (50 nM, 100 nM and 200 nM), plasma and feces samples (100 nM, 200 nM and 400 nM) were analyzed in six-fold to determine the LLOQ and ULOQ. Only feces samples were diluted 10,000x before further analysis.

Compound	Concentration (nM)	Plasma		Urine		Feces	
		Average RSD (%)	Average DEV (%)	Average RSD (%)	Average DEV (%)	Average RSD (%)	Average DEV (%)
Curcumin	1	33.5	11.2	61.4	−43	30.3	−6.6
	2	12.7	11.5	10.1	−27.9	11.8	0.6
	5	8.5	−2.1	8.1	−9.1	15.7	−3.5
	50	n.d.	n.d.	9.4	1	n.d.	n.d.
	100	8.5	−2.1	7.6	11.6	3.7	9.3
	200	4.1	10.2	15.2	10.9	3.9	18.3
	400	8.6	12	n.d.	n.d.	4.3	10.7
Demethoxy-curcumin	1	22.9	−6.5	24.7	62.2	8.2	9.6
	2	17.2	12.6	16.8	14.4	12.6	3.4
	5	5.1	−0.6	23.9	−16.6	9.7	4.6
	50	n.d.	n.d.	14.8	−9.3	n.d.	n.d.
	100	8.4	−1.8	15.0	−1.2	3.2	0.0
	200	7.5	6.4	3.8	−7.8	3.9	−3.4
	400	8.1	−4.6	n.d.	n.d.	2.6	−6.2
Bisdemethoxy-curcumin	1	19.5	−11.4	17.1	40.6	n.d.	n.d.
	2	23.6	5.0	25.6	−3.5	25.3	−28.3
	5	6.4	−2.7	14.8	−3.0	12.2	−2.4
	50	n.d.	n.d.	14.3	−10.0	n.d.	n.d.
	100	7.0	3.0	16.3	0	4.5	5.6
	200	6.0	2.2	14.7	−10.9	5.7	−0.2
	400	11.5	−3.4	n.d.	n.d.	2.5	−4.9
Tetrahydro-curcumin	1	16.4	−4.1	No peak	No peak	5.2	17.6
	2	19.1	7.0	16.2	7.4	10.4	6.6
	5	3.5	−2.7	14.7	−9.6	8.8	2.5
	50	n.d.	n.d.	10.6	−0.3	n.d.	n.d.
	100	6.7	5.5	13.7	3.7	3.5	−1.3
	200	3.4	5.7	15.8	−7.0	2.8	−2.2
	400	6.9	2.7	n.d.	n.d.	2.4	−2.6
Piperine	1	n.d.	n.d.	28.3	2.3	15.1	−28.5
	2	13.3	−3.2	50.0	−21.1	8.8	−13.6
	5	5.0	2.2	10.2	−6.2	11.1	−3.4
	10	4.9	7.4	n.d.	n.d.	n.d.	n.d.
	50	n.d.	n.d.	4.9	6.4	n.d.	n.d.
	100	6.1	−0.3	12.8	11.0	2.8	−0.8
	200	3.7	0.0	13.5	3.3	5.0	−0.6
	400	4.6	−6.6	n.d.	n.d.	2.8	−10.7

The ULOQ was validated in plasma for curcumin demethoxycurcumin, bisdemethoxycurcumin, tetrahydrocurcumin and piperine at 400 nM for all compounds. The ULOQ in urine was validated on 200 nM for all compounds. Finally, for the diluted feces samples the ULOQ was determined on 400 nM for all compounds.

### 3.5 Linearity

All compounds in plasma can be quantified on a linear range of 2–400 nM (Table 4). Urine samples can be quantified for curcumin, demethoxycurcumin, bisdemethoxycurcumin, tetrahydrocurcumin and piperine in the linear range of 2–400 nM, 2–400 nM, 2–200 nM, 10–400 nM and 2–400 nM, respectively (Table 4). Finally, the feces samples can be quantified for curcumin, demethoxycurcumin, bisdemethoxycurcumin, tetrahydrocurcumin and piperine in the linear range of 2–200 nM, 2–200 nM, 5–200 nM, 5–200 nM and 5–200 nM, respectively (Table 4). None of the compounds showed any signs for a constant translational or rotational bias in all of the different matrixes.

**Table 4:** Summary of linearity of curcumin, demethoxycurcumin, bisdemethoxycurcumin, tetrahydrocurcumin and piperin-135 in plasma, urine and feces. A calibration line was measured over three runs over three days. For each line the power value was established by weight estimated regression analysis in SPSS.

Compound	Matrix	Concentration Range (nM)	Weight	LOF	Systematic error	Proportional error	Outcome
Curcumin	Plasma	2–400	2	No	No	No	Linear
	Urine	2–400	2	No	No	No	Linear
	Feces	2–200	2	No	No	No	Linear
Demethoxy-curcumin	Plasma	2–400	2	No	No	No	Linear
	Urine	2–400	1	No	No	No	Linear
	Feces	2–200	2	No	No	No	Linear
Bisdemethoxy-curcumin	Plasma	2–400	2	No	No	No	Linear
	Urine	2–200	2	No	No	No	Linear
	Feces	5–200	2	No	No	No	Linear
Tetrahydro-curcumin	Plasma	2–400	2	No	No	No	Linear
	Urine	10–200	2	No	No	No	Linear
	Feces	5–200	2	No	No	No	Linear
Piperin-135	Plasma	2–400	2	No	No	No	Linear
	Urine	2–400	2	No	No	No	Linear
	Feces	5–200	2	No	No	No	Linear

### 3.6 Recovery and ionization loss

Plasma, urine and feces recovery of curcumin was respectively at 96.7 ± 16.7%, 74.1 ± 7.6% and 109.2 ± 38.5% (Table 5). Overall, all compounds showed a lower recovery and higher ion suppression in urine than compared to plasma and feces recovery and ion suppression. The use of deuterated internal standards is an effective means to minimize the effect of ion suppression and recovery and thus achieving more accurate results.

**Table 5:** Summery of recovery and ionization loss in plasma, urine and feces. The recovery of the sample pretreatment was calculated from the ratio of the slopes of the pre-pretreatment spike over the post-pretreatment spiked samples. The ratio of the slopes of the post-pretreatment spike over the unprocessed academic calibration line resulted in thematrix effect by the biological matrix.

	Recovery			Matrix effect		
	Plasma	Urine	Feces	Plasma	Urine	Feces
Compound	Average ± SD (%)	Average ± SD (%)	Average ± SD (%)	Average ± SD (%)	Average ± SD (%)	Average ± SD (%)
Curcumin	96.7 ± 16.7	74.1 ± 7.6	109.2 ± 38.5	12.9 ± 7.5	27.6 ± 21.8	15.8 ± 1.7
Demethoxycurcumin	96.8 ± 17.0	73.4 ± 16.2	106.2 ± 48.7	3.5 ± 10.3	49.9 ± 17.1	27.8 ± 15.7
Bisdemethoxycurcumin	101.5 ± 17.0	64.3 ± 13.5	111.1 ± 52.4	17.8 ± 2.6	56.3 ± 19.9	24.1 ± 24.2
Tetrahydrocurcumin	102.9 ± 28.0	101.5 ± 17.7	113.4 ± 25.2	−12.1 ± 46.1	51.6 ± 16.5	15.8 ± 8.9
Piperine-135	109.3 ± 0.8	101.2 ± 20.4	109.3 ± 44.7	4.7 ± 21.2	38.5 ± 15.1	5.5 ± 24.8

### 3.7 Accuracy and precision

All compounds showed acceptable within-day or between-day variability in plasma, urine or diluted feces (Table 6, Table 7, Table 8). Furthermore, all compounds demonstrated concentrations within 15% deviation from the nominal concentration.

**Table 6:** Summary of accuracy and precision of compounds in plasma. The accuracy was determined by dividing the mean observed concentration by the nominal concentration. The root of mean square within groups was divided by the total mean to obtain the within-day precision. The between-day precision was calculated by dividing the difference between mean square between groups by mean square within groups by six, the root of this number was divided by the total mean.

Compound	Concentration (nM)	Day 1 (%)	Day 2 (%)	Day 3 (%)	within day variability (%)	Between day variability (%)
Curcumin	100	94.5	92.7	98.4	4.4	2.5
	50	93.9	91.5	98.4	6.4	2.6
	10	90.7	92.2	98.3	3.4	4.2
Demethoxycurcumin	100	99.3	90.9	89.5	3.2	5.5
	50	105.3	98.6	95.9	3.0	4.6
	10	102.8	97.7	93.4	3.6	4.6
Bisdemethoxycurcumin	100	99.2	90.7	88.4	3.8	5.9
	50	104.5	96.2	89.5	3.9	7.3
	10	104.1	101.1	92.5	5.2	5.7
Tetrahydrocurcumin	100	93.4	92.5	99.8	1.9	4.1
	50	95.5	95.6	102.6	3.1	3.9
	10	91.8	93.6	100.9	1.9	5.2
Piperine-135	100	100.9	95.8	88.5	4.6	2.3
	50	98.3	94.6	83.2	5.2	0.2
	10	92.6	96.8	87.3	5.7	1.2

**Table 7:** Summary of accuracy and precision of compounds in urine. The accuracy was determined by dividing the mean observed concentration by the nominal concentration. The root of mean square within groups was divided by the total mean to obtain the within-day precision. The between-day precision was calculated by dividing the difference between mean square between groups by mean square within groups by six, the root of this number was divided by the total mean. *: No statistically significant additional spread was found as a result of performing the analysis in different runs.

Compound	Concentration (nM)	Day 1 (%)	Day 2 (%)	Day 3 (%)	Inbetween day variability (%)	Between day variability (%)
Curcumin	200	105.8	98	110.3	6.2	2.9
	100	101.0	100.9	109.3	4.3	1.9
	50	93.7	98.6	100.8	7.0	2.8
Demethoxycurcumin	200	96.0	95.8	85.9	4.2	*
	100	102.6	103.0	90.6	8.8	4.8
	50	97.7	103.6	85.8	4.2	8.6
Bisdemethoxycurcumin	200	99.7	88.8	76.5	4.3	5.7
	100	103.1	91.9	79.8	8.7	4.7
	50	97.6	89.9	75.9	6.5	5.6
Tetrahydrocurcumin	200	94.4	98.2	96.8	4.4	3.6
	100	98.4	99.1	95.8	7.5	*
	50	99.1	95.5	92.5	4.3	0.6
Piperine-135	200	97.8	93.4	107.8	6.6	*
	100	99.7	102.6	109.5	7.0	*
	50	100.9	101.8	108.3	4.8	*

**Table 8:** Summary of accuracy and precision of compounds in diluted feces. The accuracy was determined by dividing the mean observed concentration by the nominal concentration. The root of mean square within groups was divided by the total mean to obtain the within-day precision. The between-day precision was calculated by dividing the difference between mean square between groups by mean square within groups by six, the root of this number was divided by the total mean. *: No statistically significant additional spread was found as a result of performing the analysis in different runs.

Compound	Concentration (nM)	Day 1 (%)	Day 2 (%)	Day 3 (%)	Inbetween day variability (%)	Between day variability (%)
Curcumin	200	103.3	116.2	90.7	10.6	9.5
	100	102.7	113.5	112.3	8.0	5.2
	50	99.2	108.9	101.8	3.6	4.6
Demethoxycurcumin	200	89.6	98.8	101.4	6.9	5.8
	100	92.4	98.9	97.7	7.2	4.6
	50	92.8	100.7	94.7	2.5	4.1
Bisdemethoxycurcumin	200	91.2	104.7	109.7	13.2	7.7
	100	95.3	108.0	97.9	6.8	10.1
	50	96.3	113.7	94.7	3.6	10.3
Tetrahydrocurcumin	200	95.3	103.5	102.8	4.6	4.1
	100	96.4	101.0	101.1	7.0	4.0
	50	95.2	100.5	97.2	3.3	2.4
Piperine-135	200	96.1	103.8	93.7	4.7	5.0
	100	100.3	103.2	106.1	7.4	1.9
	50	99.5	101.5	100.7	3.2	*

### 3.8 Stability

Thawing the sample resulted in a minimal loss of concentration in comparison to the nominal concentration of 100 nM (Table 9). Table 10 shows the results of the stability of the different compounds in different conditions, namely on ice in a dark environment, at room temperature in a dark environment and at room temperature in a light environment.

**Table 9:** Summary of the stability of curcumin, demethoxycurcumin, bisdemethoxycurcumin, tetrahydrocurcumin and piperine-135 in DMSO. Freeze-thaw stability of QC stock solution 10 μM at −20 °C was performed with stock solution that had underwent 0, 1 and 10 freeze-thaw cycles.

Concentration and number of times thawing	Curcumin	Demethoxy-curcumin	Bisdemethoxy-curcumin	Tetrahydro-curcumin	Piperine-135
QC 100 nM 0x thawed	100%	100%	100%	100%	100%
QC 100 nM 1x thawed	99.8%	102.2%	100.7%	101.0%	101.8%
QC 100 nM 10x thawed	96.8%	103.1%	97.1%	100.0%	101.7%

**Table 10:** Results of curcumin, demethoxycurcumin, bisdemethoxycurcumin, tetrahydrocurcumin and piperine-135 storage in different conditions.

Stability sample during storage	Concentration	AS	On ice - dark	Room temperature - dark	Room temperature - light
Curcumin	QC 10	100%	76%	83%	82%
	QC 50	100%	89%	94%	91%
	QC 100	100%	87%	90%	89%
Demethoxy-curcumin	QC 10	100%	93%	95%	87%
	QC 50	100%	96%	100%	93%
	QC 100	100%	91%	95%	89%
Bisdemethoxy-curcumin	QC 10	100%	90%	95%	88%
	QC 50	100%	90%	92%	89%
	QC 100	100%	84%	84%	82%
Tetrahydrocurcumin	QC 10	100%	96%	89%	88%
	QC 50	100%	99%	93%	93%
	QC 100	100%	94%	88%	87%
Piperine-135	QC 10	100%	77%	78%	77%
	QC 50	100%	90%	85%	90%
	QC 100	100%	89%	85%	85%

### 3.9 Applicability of the method

**Fig. 4:**
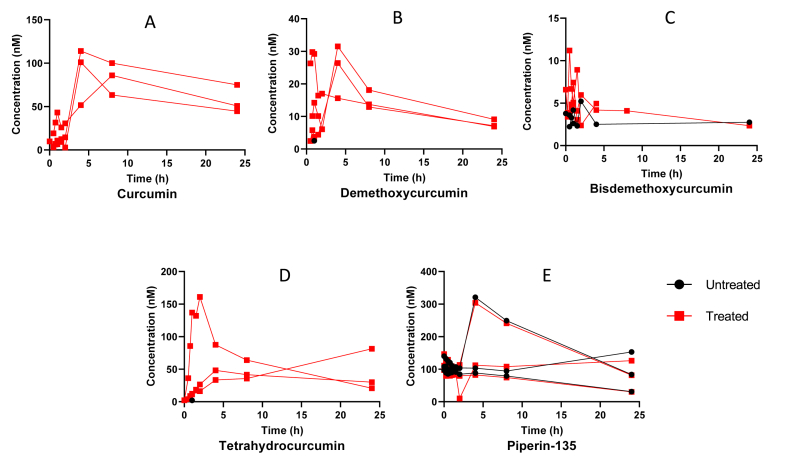
Applicability of the method showing the plasma concentration curve of curcumin (A), demethoxycurcumin (B), bisdemethoxycurcumin (C), tetrahydrocurcumin (D) and piperin-135 (E) in three volunteers intake of 4 capsules containing 600 mg curcuminoids (95% purity). Untreated means no addition of β-glucuronidase where treated means the addition of β-glucuronidase to quantify unconjugated forms.

**Fig. 5:**
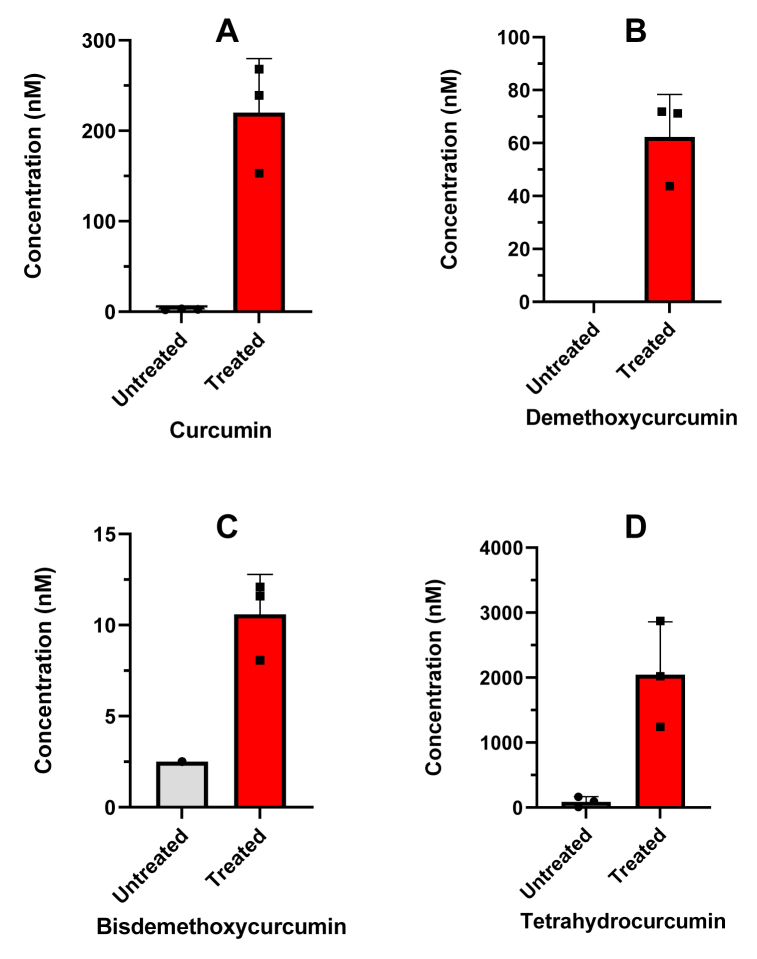
Applicability of the method showing the 24h urine concentration of curcumin (A), demethoxycurcumin (B), bisdemethoxycurcumin (C) and tetrahydrocurcumin (D) in three volunteers after intake of 4 capsules containing 600 mg curcuminoids (95% purity). Untreated means no addition of β-glucuronidase where treated means the addition of β-glucuronidase to quantify unconjugated forms.

**Fig. 6:**
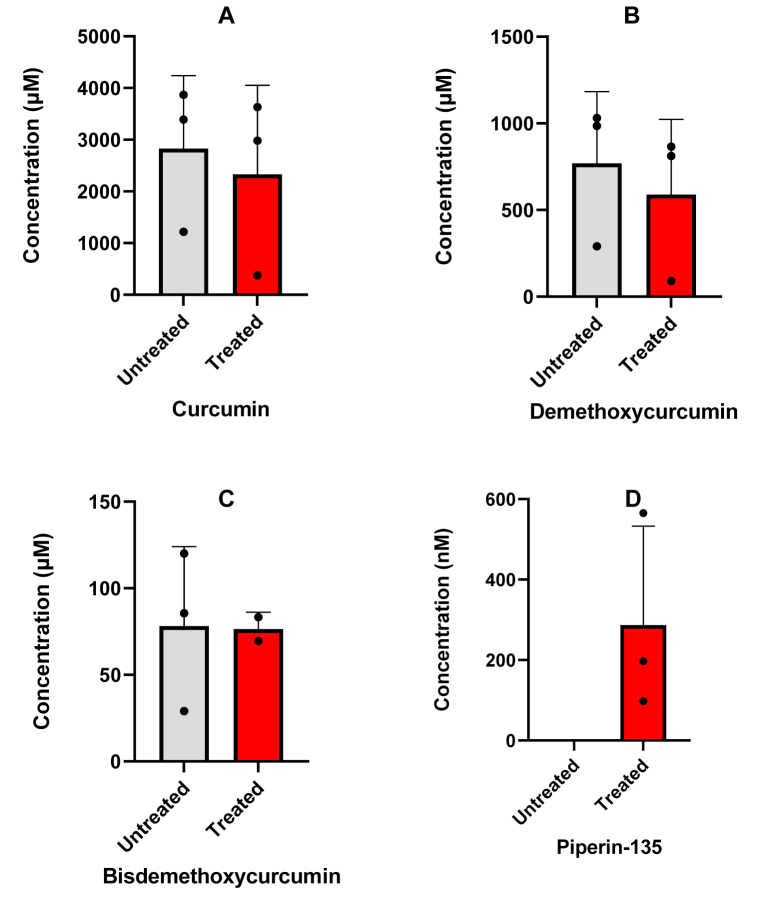
Applicability of the method showing the 48h feces concentration of curcumin (A), demethoxycurcumin (B), bisdemethoxycurcumin (C) and piperin-135 (D) in three volunteers after intake of 4 capsules containing 600 mg curcuminoids (95% purity). Untreated means no addition of β-glucuronidase where treated means the addition of β-glucuronidase to quantify unconjugated forms.

Fig. 4 shows the plasma concentration of curcumin (Fig. 4A), demethoxycurcumin (Fig. 4B), bisdemethoxycurcumin (Fig. 4C), tetrahydrocurcumin (Fig. 4D) and piperine (Fig. 4E) in three participants. All compounds, except piperine, show plasma levels below our validated range of 2 nM. After treatment with β-glucuronidase an increase in the plasma peak concentrations of curcumin, demethoxycurcumin, bisdemethoxycurcumin and tetrahydrocurcumin is observed, however plasma concentrations remain low. Fig. 5 shows the urine concentrations of curcumin (Fig. 5A), demethoxycurcumin (Fig. 5B), bisdemethoxycurcumin (Fig. 5C) and tetrahydrocurcumin (Fig. 5D) of the same participants as Fig. 4. After treatment with β-glucuronidase an increase in the urine concentrations of curcumin, demethoxycurcumin, bisdemethoxycurcumin and tetrahydrocurcumin is observed as can be expected after conjugation in the liver. Fig. 6 shows feces concentrations of curcumin (Fig. 6A), demethoxycurcumin (Fig. 6B), bisdemethoxycurcumin (Fig. 6C) and piperine (Fig. 6D) between 50 and 4000 μM. The percentage of curcumin in feces is roughly 21.7% of the total expected dosage, whereas the percentage of demethoxycurcumin and bisdemethoxycurcumin amounted to respectively 24.7% and 13% of the total expected dosage. No tetrahydrocurcumin was found as this was mainly excreted via the urine. Piperine concentrations were still found in the feces of one participant despite the fact that all participants had diet restrictions to avoid piperine in take. This is however difficult to achieve since many processed foods have traces of piperine.

## 4 Discussion

In this paper we present the development and validation of an HPLC-MS/MS method for the quantification of curcumin, demethoxycurcumin, bisdemethoxycurcumin, tetrahydrocurcumin and piperine. All curcumin analogs and piperine were quantified in a single analytical run with gradient elution conditions. The accuracy and precision was acceptable also due to the use of appropriate stable isotope labeled (deuterated) internal standards. The LLOQ of this method was in the low nanomolar range and is therefore suited to determine the plasma levels after oral intake to asses bioavailability of different types of curcumin formulations. The method is also applicable to urine and feces specimens, making it useful for comprehensive pharmacokinetic and mass balance studies.

**Fig. 7:**
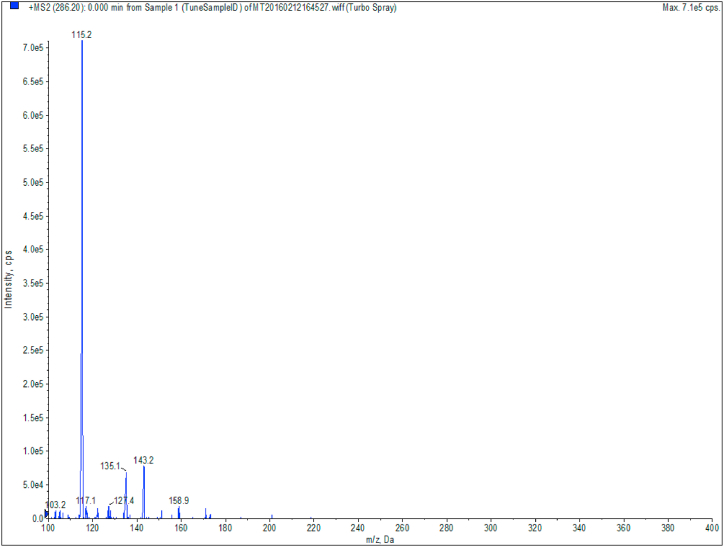
Fragmentation of Piperin in fragments with *m*/*z* 115.2, 135.1 and 143.2. Fragment 115.2 shows saturation from the column whereas piperin fragment *m*/*z* 135.1 shows a sharp peak without saturation.

To optimize our method of analysis we tested several reconstitution ratios of MeOH: H_2_O and mobile phase compositions. The reconstitution ratio MeOH: H_2_O of 60:40 and MeOH: H_2_O containing 0.1% of formic acid as mobile phase proved to be the best fit for our method. All compounds except tetrahydrocurcumin eluted in sharp peaks. It has been shown that tetrahydrocurcumin has a better peak shape when using a more basic mobile phase, e.g. 0.02% ammonium hydroxide [21]. Unfortunately, after using this basic mobile phase it appeared to negatively affect the chromatograms behavior of the other analogs, resulting in all curcuminoids to have severe front tailing as seen in Fig. 3B. We therefore kept the acidic mobile phase and explored if analyzing tetrahydrocurcumin on basis of the peak height instead of the peak area is a feasible option. All other compounds were analyzed on peak area. The duration and ratio of the gradient was optimized to allow all peaks to have the maximum selectivity and specificity possible. We used TBME as organic solvent since samples pretreated with ethylacetate and diethyl ether showed higher ion suppression and lower recovery (data not shown). We initially used fresh-frozen plasma from healthy volunteers, but this consistently contained relatively high piperine peaks in blank samples. Fortunately, pooled omniplasma showed no peaks in the chromatogram of blank samples. Omniplasma is pooled processed plasma which is inactivated for viruses, parasites, bacteria and compounds leading to allergy [22]. During this inactivation process, piperine seems to be removed. During the optimization of our MS/MS method, we noted that piperine forms several product ions when fragmented of which *m*/*z* 115.2 gave the highest response. However, using this fragment resulted in saturated peaks where the top of the peak was flattened. We, therefore, selected the less intense product ion *m*/*z* 135, which resulted in a more similar peak intensity compared to the other analytes (see Fig. 7).

Finally, the conditions for using β-glucuronidase was assessed in order to apply the method in a clinical setting. β-glucuronidase hydrolyses conjugated curcumin allowing unconjugated curcumin to form [23,24]. Many supplement manufactures of curcumin only report the curcumin plasma levels after β-glucuronidase incubation. Consequently, it remains unclear if the alternative formulation really increase the levels of unconjugated curcumin after oral intake. We assessed that de-conjugation by β-glucuronidase was complete after incubating curcumin-glucuronide for 1 h at 37 °C using a β-glucuronidase concentration of 20 IU/μL. Prolonging the incubation did not result in more unconjugated curcumin.

The intra- and inter-day accuracy and specificity of all analytes were evaluated at concentrations of 10, 50 and 100 nM in plasma and 50, 100 and 200 nM in urine and diluted feces. Similar to Heath et al., Vijaya Saradhi et al. and Cao et al. we made use of an acidic mobile phase leading to reduced sensitivity of tetrahydrocurcumin which can be shown by the peak tailing/fronting [14,25,26].

Unlike some of the other published curcumin quantifying methods, our use of deuterated isoforms of the analytes curcumin, bisdemethoxycurcumin, tetrahydrocurcumin and piperine allowed for an optimal correction in recovery and ion suppression due to the similar characteristics of their matching compounds. Using stable isotope-labeled (deuterated) internal standard provide the best correction for loss of analytes during the sample extraction and loss of signal due to ion-suppression and greatly improves the robustness of the assay.

The lowest limit of quantification (LLOQ) defines the sensitivity of the method. Piperine plasma concentrations are considerably higher than curcumin and analogs. Thus a higher LLOQ for piperine does not impact the usefulness. There are many curcumin formulations on the market that contain piperine as add-on. The ability to measure the concentration of piperine in various matrixes simultaneously with the curcuminoids and its metabolites is a clear benefit of our method. Concentrations of all curcuminoids are expected to be much higher in feces. The LLOQ in diluted feces is therefore higher than in plasma and urine.

The linearity of tetrahydrocurcumin was between 10 and 200 nM in urine. Tetrahydrocurcumin is a metabolite of curcumin. Concentrations of tetrahydrocurcumin in urine are however expected to be higher than 10 nM due to the metabolism that takes place in the liver. Therefore, linearity of curcumin in feces was calculated between 2 and 200 nM. Samples out this validated range will be diluted, which will also decrease any matrix effects of feces on our compounds of interest.

Table 5 shows that the recovery in plasma and feces was near 100%. Feces samples were however diluted by a factor 10.000x to account for the high concentrations found in feces and for the high level of ion suppression. This 10.000x dilution subsequently caused the loss of any fibers and other interfering compounds to interact with the curcuminoids and its metabolites what would result in the lower recovery and ion suppression. Urine samples however showed a recovery of 74.1 ± 7.6% and ion suppression of 27.6 ± 21.8%. This is significantly lower compared to feces and plasma samples. This may be due to the fact that urine is in this case a biological waste material that is not diluted like the feces samples. It can still contain breakdown products of foods, drinks, endogenous waste metabolites and bacterial by-products. These could have an interaction with curcumin and other curcuminoids to lower the recovery and increase the ion suppression [27].

All compounds showed that the within-day and between day variability was acceptable. All compounds are stable in DMSO. Thawing and freezing of the stock solutions showed no significant impact on the stability of curcumin. The stability of the compounds in the biological matrix plasma showed that when left on ice the concentration decreased. This could be explained by the fact that the solubility of curcuminoids decreases at lower temperatures. As has been intensively described in the literature, light has a severe impact on the stability of curcumin. This is due to the chromophore characteristics of curcumin and therefore its photo(oxidative) degradation [28]. All compounds are proven to be more stable at room temperature in a dark environment.

## 5 Conclusion

A HPLC-MS/MS method was developed and validated for the simultaneous quantification of curcumin, demethoxycurcumin, bisdemethoxycurcumin, tetrahydrocurcumin and piperine in human plasma, urine or feces using their deuterated forms as internal standards. This method can be used to analyze the bioavailability of curcumin formulations in order to critically verify claims being made by curcumin manufacturers and helps us to provide insight in the true bioavailability of curcumin supplements.

## Author contribution statement

M.A.G.M. Kroon, PharmD.: Conceived and designed the experiments; Performed the experiments; Analyzed and interpreted the data; Contributed reagents, materials, analysis tools or data; Wrote the paper.

H.W.M. van Laarhoven, Prof. Dr.; E.L. Swart, Prof. Dr.; E.M. Kemper, Dr.: Analyzed and interpreted the data; Wrote the paper.

O. van Tellingen: Conceived and designed the experiments; Analyzed and interpreted the data; Contributed reagents, materials, analysis tools or data; Wrote the paper.

## Data availability statement

Data will be made available on request.

## Declaration of interest’s statement

The authors declare no competing interests.

## Additional information

No additional information is available for this paper.
